# Synthesis and mesomorphic properties of calamitic malonates and cyanoacetates tethered to 4-cyanobiphenyls

**DOI:** 10.3762/bjoc.8.40

**Published:** 2012-03-09

**Authors:** Katharina C Kress, Martin Kaller, Kirill V Axenov, Stefan Tussetschläger, Sabine Laschat

**Affiliations:** 1 Institut für Organische Chemie, Universität Stuttgart, Pfaffenwaldring 55, 70569 Stuttgart, Germany

**Keywords:** cyanoacetates, 4-cyanobiphenyls, liquid crystals, malonates, nematic

## Abstract

4-Cyano-1,1'-biphenyl derivatives bearing ω-hydroxyalkyl substituents were reacted with methyl 3-chloro-3-oxopropionate or cyanoacetic acid, giving liquid-crystalline linear malonates and cyanoacetates. These compounds formed monotropic nematic phases at 62 °C down to ambient temperature upon cooling from the isotropic liquid. The mesomorphic properties were investigated by differential scanning calorimetry, polarizing optical microscopy and X-ray diffraction (WAXS).

## Introduction

Nematic liquid crystals display mesophases in which the molecules are oriented along one vector defined by the director axis, but with the molecular arrangement in random positional order [1]. Nematic phases typically display low viscosity [2–4]. Due to the long-range orientational order they reveal anisotropic properties. These features make nematic liquid crystals very attractive materials for electronics [5–8], for the construction of liquid crystal displays [9–11], or as anisotropic conductors [12–13]. Over the past few decades, a huge variety of organic calamitic compounds, which form nematic liquid crystals, have been synthesized and investigated [1,14].

**Scheme 1:**
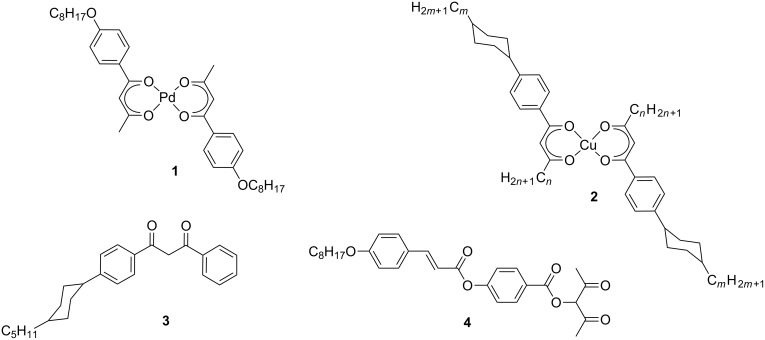
Diketonato metallomesogens and diketones with mesomorphic properties.

Bulkin et al. were the first to investigate the phase behaviour of metal β-diketonate complexes such as **1** [15] (Scheme 1). Although they were not able to detect any mesophases, their study motivated others to examine the mesomorphic properties of β-diketonates in more detail [16]. Among the first examples of a nematic β-diketonate is the Cu complex **2** described by Haase [17–21]. In contrast to the various diketonato metallomesogens only a little information is available about the mesomorphic properties of metal-free diketones. Among the few examples are the nematic compounds **3** [22] and **4** [23–24] (Scheme 1).

**Scheme 2:**
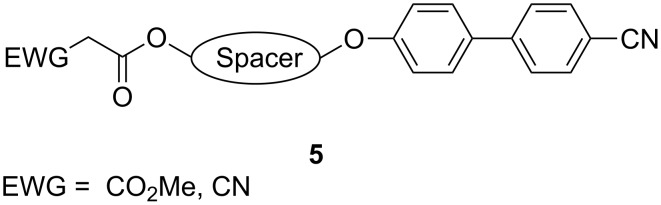
Malonates and cyanoacetates tethered to calamitic 4-cyanobiphenyl units.

The corresponding malonates and cyanoacetates are well known as suitable ligands for strong coordination of main-group and transition metals [25]. Benzylidene derivatives of malonic esters, so called swallow-tailed liquid crystals, were described as forming smectic phases [26]. However, most work on liquid-crystalline malonates has been devoted to C_60_ fullerene dendrimers [27–31]. Only a few liquid crystalline cyanoacetates have been described so far. The first example, a dihydrazide, was reported by Schubert [32]. Furthermore some calamitic and bent-core mesogens derived from α-cyanocinnamic acid were described in the literature [33–34]. Therefore, we decided to explore the synthesis and mesomorphic properties of malonates and cyanoacetates **5** tethered to calamitic 4-cyanobiphenyl units (Scheme 2).

## Results and Discussion

**Scheme 3:**
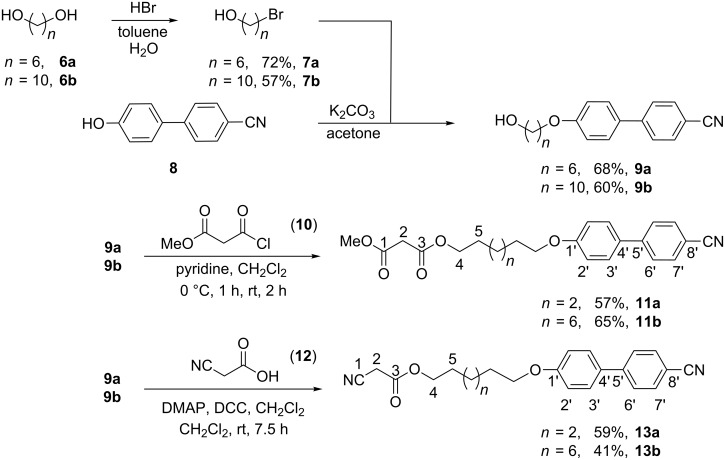
Synthesis of malonate and cyanoacetates tethered to 4-cyano-biphenyl moieties.

The syntheses of malonate and cyanoacetate derivatives **11**, **13** are shown in Scheme 3. Starting from the corresponding diols **6a**,**b**, 6-bromohexan-1-ol (**7a**) and 10-bromodecan-1-ol (**7b**) were obtained in moderate yields by bromination with aqueous HBr in toluene [35]. The bromides **7a**,**b** were reacted with 4-cyano-1,1'-biphenol (**8**) in acetone in the presence of K_2_CO_3_ giving compounds **9a**,**b**, bearing C_6_- or C_10_-spacers, in 68% and 60% yield, respectively [36–38] after recrystallization from methanol (Scheme 3).

The malonate unit was attached by treatment of the compounds **9a**,**b** with methyl 3-chloro-3-oxopropionate (**10**) in the presence of pyridine in CH_2_Cl_2_ to yield the malonates **11a**,**b** in 57% and 65%, respectively, after column chromatography. In a parallel approach, the precursors **9a**,**b** were converted to the corresponding cyanoacetates **13a**,**b** by esterification of cyanoacetic acid (**12**) in the presence of DMAP and dicyclohexylcarbodiimide in CH_2_Cl_2_. After chromatography the cyanoacetates **13a**,**b** were isolated as colourless solids in 59% and 41% yield.

**Figure 1:**
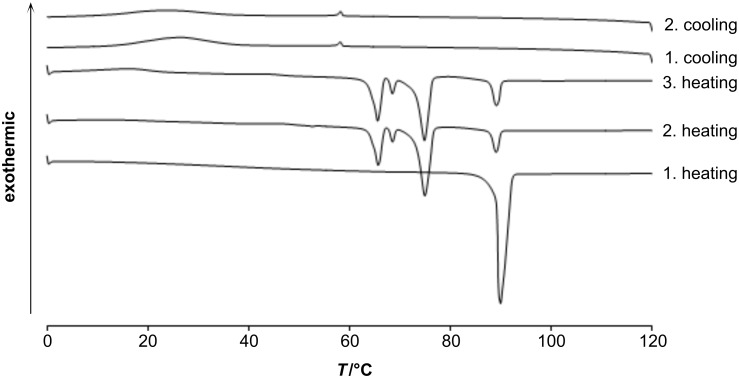
DSC traces of **13a** (heating/cooling rate 5 K/min).

**Figure 2:**
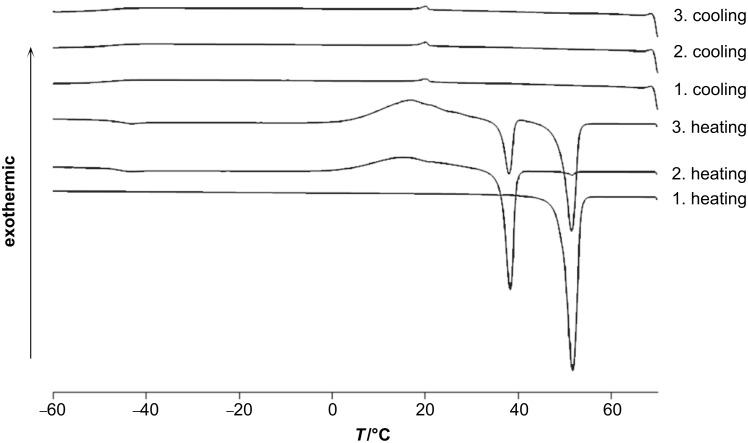
DSC traces of **11a** (heating/cooling rate 10 K/min).

The obtained malonate and cyanoacetic esters **11a,b** and **13a,b** were subjected to differential scanning calorimetry (DSC) studies (Figure 1, Figure 2, and Table 1).

**Table 1:** Phase-transition temperatures [°C] and enthalpies [kJ/mol] of **11** and **13**.^a^

	*n*	Cr_1_	*T*	Δ*H*	Cr_2_	*T*	Δ*H*	Cr_3_/N	*T*	Δ*H*	I	

**11a**	6	●	49.1	32.4	–	–	–	–	–	–	●	1. heating^b^
		●	–	–	–	–	–	N	21.2	−0.32	●	1. cooling^b^
		●	4.1	−12.0	●	36.2	17.6	Cr_3_	49.9	0.27	●	2. heating^b^
		●	–	–	–	–	–	N	20.9	−0.29	●	2. cooling^b^
		●	5.0	−18.2	●	35.9	7.72	Cr_3_	48.8	20.7	●	3. heating^b^
**11b**	10	●	63.4	53.2	–	–	–	–	–	–	●	1. heating^c^
		●	10.0	−24.2	●	14.8	−3.45	N	35.6	−0.68	●	1. cooling^c,d^
		●	54.8	39.8	–	–	–	Cr_3_	59.9	7.35	●	2. heating^c^
		●	14.8	−24.2	●	31.1	−0.33	N	35.4	−0.46	●	2. cooling^c^
**13a**	6	●	89.0	39.6	–	–	–	–	–	–	●	1. heating^c^
		●	–	–	–	–	–	N	58.7	−0.38	●	1. cooling^c^
		●	64.1	9.19	●	73.0	22.7	Cr_3_	87.8	−4.34	●	2. heating^c,e^
		●	–	–	–	–	–	N	58.7	−0.41	●	2. cooling^c^
		●	63.9	11.6	●	72.9	19.8	Cr_3_	87.8	5.48	●	3. heating^c,f^
**13b**	10	●	93.2	49.1	–	–	–	–	–	–	●	1. heating^c^
		●	52.7	−41.1	–	–	–	N	61.8	−0.71	●	1. cooling^c^
		●	74.7	13.5	●	86.3	−7.41	Cr_3_	91.5	41.5	●	2. heating^c,g^
		●	52.7	−41.1	–	–	–	N	61.9	−0.98	●	2. cooling^c^

^a^Cr crystalline; N nematic; I isotropic; ● phase was observed; – phase was not observed. ^b^Heating and cooling rate: 10 K/min. ^c^Heating and cooling rate: 5 K/min. ^d^Another crystal-to-crystal transition (31.2 °C, −0.24 kJ/mol) was observed. ^e^Another crystal-to-crystal transition (67.7 °C, 1.62 kJ/mol) was observed. ^f^Another crystal-to-crystal transition (67.6 °C, 1.86 kJ/mol) was observed. ^g^Another crystal-to-crystal transition (78.1 °C, 21.0 kJ/mol) was observed.

During the first heating runs all compounds did not show the appearance of any liquid-crystalline phase, but melted without decomposition into isotropic liquids. It was observed that the melting points increased with an increase of the spacer length between 4-cyanobiphenyl and ester groups. Thus, melting points were recorded at 49.1 °C/63.4 °C for the series of **11a**/**11b** and at 89.0 °C/93.2 °C for the series of **13a**/**13b**, respectively (Table 1). The cyano group is a stronger electron-acceptor than the ester function, and thus the cyanoacetic ester molecules are more polarized than the corresponding malonates. Stronger dipole–dipole interactions for cyano esters **13** led to an increase of their clearing points compared with malonates **11**. The additional Cr→Cr transitions in the 2nd and 3rd heating curves (Figure 1 and Figure 2) are probably due to equilibration and the presence of keto-enol tautomers. Molecular geometry phase-behaviour relationships in keto-enamine/imino-enol tautomers of ferrocenophanes have been previously studied by Galyametdinov [39]. In the first cooling runs the appearance of nematic mesophases was observed for both series **11a**,**b** and **13a**,**b**. All compounds displayed small transition enthalpies in a range between −0.3 and −0.7 kJ/mol (Table 1) for the transition from the isotropic liquid to the corresponding mesophases. While C_6_-linked compounds displayed monotropic nematic mesophases at temperatures of 21 °C for **11a** and 59 °C for **13a**, their longer and more flexible C_10_-linked homologues showed higher transition temperatures at 36 °C for **11b** and 62 °C for **13b** upon cooling from the isotropic liquid. Due to supercooling, no crystallisation could be observed for compound **11a** and, therefore, no mesophase range could be determined. But the second and third heating runs of **11a** showed broad recrystallization peaks (Figure 2).

### Polarizing optical microscope (POM) studies

**Figure 3:**
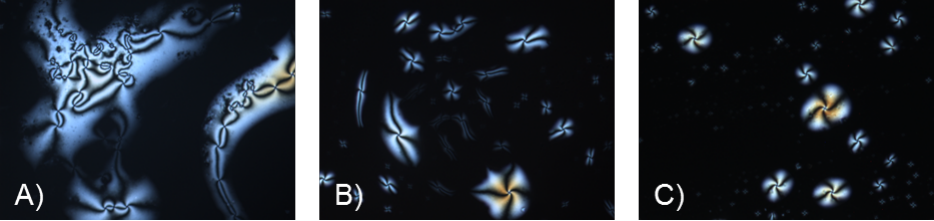
Schlieren textures of **11a** and **11b** under crossed polarizers, upon cooling (cooling rate 5 K/min) from the isotropic liquid (magnification 200×): (A) **11a** (20 °C), (B) **11a** (14 °C), (C) **11b** (29 °C).

**Figure 4:**
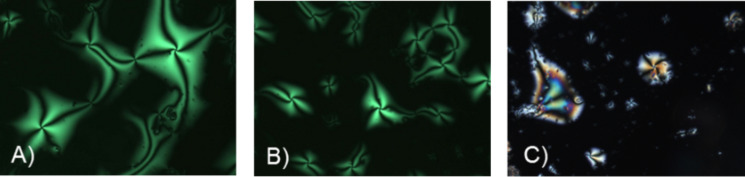
Schlieren textures of **13a** and **13b** under crossed polarizers upon cooling (cooling rate 5 K/min) from the isotropic liquid (magnification 200×): (A) **13b** (61 °C), (B) **13b** at 61 °C, different section, (C) **13a** (46 °C).

POM observations of compounds **11a**,**b** and **13a**,**b** revealed textures typical for nematic phases, only upon cooling from the isotropic liquid (Figure 3 and Figure 4).

Schlieren textures with fourfold brushes were observed for compound **13b** at the transition from the isotropic liquid into the nematic phase. Quite similar textures were published by Dierking [40–41]. The areas without birefringence in Figure 3 and Figure 4 derive from homeotropic alignment of the molecules.

### X-ray diffraction studies

**Figure 5:**
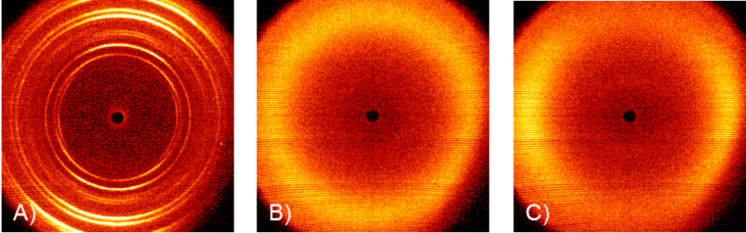
2D X-ray scattering patterns of **11a**: (A) crystalline phase at 50 °C, (B) isotropic phase at 25 °C, and (C) nematic phase at 15 °C.

The assignment of the nematic mesophases were exemplarily confirmed by wide-angle X-ray scattering (WAXS) experiments on compound **11a**. Representative 2D diffractograms of the crystalline phase, the isotropic phase and the nematic phase are shown in Figure 5.

In the isotropic phase (Figure 5, part B) only a diffuse symmetric halo is observed. The diffraction pattern of **11a** at 15 °C (Figure 5, part C) displays a halo split into two diffuse, crescent reflections, which is typical for nematic mesophases [42].

## Conclusion

The mesogenic 4-cyano-1,1'-biphenyl group can be attached to either a malonate or a cyanoacetic ester scaffold by means of simple reaction sequences and with the aid of cheap chemical precursors. Linked ester molecules **11a**,**b** and **13a**,**b** have a distinct linear shape and easily form monotropic mesophases at ambient temperature upon cooling from the isotropic liquid. Following POM and X-ray studies, nematic mesophases could be assigned to all the described compounds **11a**,**b** and **13a**,**b**.

## Experimental

### General information

All reactions were carried out under a nitrogen atmosphere with Schlenk-type glassware. Solvents were dried and distilled under nitrogen prior to use. Flash chromatography was performed on silica gel, with grain size 40–63 μm (Macherey-Nagel).

The following instruments were used for physical characterization of the compounds. Elemental analyses: Carlo Erba Strumentazione Elemental Analyzer, Modell 1106. NMR: Bruker ARX-500 (^1^H, 500 MHz; ^13^C, 125 MHz). Assignments of the resonances are supported by 2D experiments and chemical shift calculations. ^1^H and ^13^C NMR spectra were referenced to an internal Me_4_Si (TMS) standard. IR: Bruker 22 FT-IR spectrometer with a golden-gate single-reflection diamond ATR system. MS: Bruker Daltonics mikro-TOF-Q (ESIMS). Differential scanning calorimetry (DSC): Mettler-Toledo DSC 822e (heating/cooling rates were 5 or 10 K·min^−1^). Polarizing optical microscopy: Olympus BX50 polarizing microscope combined with a Linkam TP93 central controller. X-ray diffraction (WAXS): Bruker AXS Nanostar C diffractometer employing Ni-filtered Cu Kα radiation (λ = 1.5418 Å).

### 6-[(4'-cyano-[1,1'-biphenyl]-4-yl)oxy]hexyl methyl malonate (**11a**)

Pyridine (150 mg, 1.69 mmol) and then methyl 3-chloro-3-oxopropionate (**10**) (114 mg, 0.84 mmol) were added over 10 min at 0 °C under a N_2_ atmosphere to a solution of 4'-((6-hydroxyhexyl)oxy)-[1,1'-biphenyl]-4-carbonitrile (**9a**) (500 mg, 1.69 mmol) in abs. CH_2_Cl_2_ (5 mL). The reaction mixture was stirred at 0 °C for 1 h, then for 2 h at rt. The reaction was quenched with 1 N H_2_SO_4_ (3 mL). The aqueous layer was extracted with CH_2_Cl_2_ (3 × 5 mL). The combined organic layers were washed with brine (40 mL), dried over MgSO_4_ and evaporated under reduced pressure. The crude product was purified by column chromatography on silica gel (hexanes/EtOAc 20:1) to give **11a** as a colourless solid (188 mg, 0.48 mmol, 57%). Mp 49.1 °C; ^1^H NMR (500 MHz, CDCl_3_) δ 1.41–1.47 (m, 2H, 6-H), 1.49–1.55 (m, 2H, 7-H), 1.67–1.73 (m, 2H, 5-H), 1.79–1.85 (m, 2H, 8-H), 3.39 (s, 2H, 2-H), 3.74 (s, 3H, OC*H*_3_), 4.01 (t, *J* = 6.4 Hz, 2H, 9-H), 4.17 (t, *J* = 6.4 Hz, 2H, 4-H), 6.98 (d, *J* = 8.5 Hz, 2H, 2'-H), 7.52 (d, *J* = 8.5 Hz, 2H, 3'-H), 7.64 (d, *J* = 8.5 Hz, 2H, 6'-H), 7.69 (d, *J* = 8.5 Hz, 2H, 7'-H) ppm; ^13^C NMR (125 MHz, CDCl_3_) δ 25.6, 25.7 (C-6, C-7), 28.4 (C-5), 29.0 (C-8), 41.4 (C-2), 52.5 (O*C*H_3_), 65.6 (C-4), 67.8 (C-9), 110.0 (C-8'), 115.0 (C-2'), 119.1 (*C*N), 127.0 (C-6'), 128.3 (C-3'), 131.3 (C-5'), 132.6 (C-7'), 145.3 (C-4'), 159.7 (C-1'), 166.6 (C-3), 167.0 (C-1) ppm; ATR–FTIR 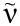: 2936 (m), 2858 (w), 2224 (m), 1967 (w), 1730 (s), 1602 (m), 1494 (m), 1246 (s), 1014 (m), 822 (s); ESIMS (*m*/*z*): 343.1 [M + K]^+^, 418.1 [M + Na]^+^, 396.1 [M + H]^+^, 278.15 [C_19_H_20_NO]^+^; Anal. calcd for C_23_H_25_NO_5_: C, 69.86; H, 6.37; N, 3.54; found: C, 69.47; H, 6.37; N, 3.47; *R*_f_ 0.56 (hexanes/EtOAc 2:1).

### 10-[(4'-cyano-[1,1'-biphenyl]-4-yl)oxy]decyl methyl malonate (**11b**)

The ester **11b** was obtained by the same procedure as described above for **11a** from 4'-((10-hydroxydecyl)oxy)-[1,1'-biphenyl]-4-carbonitrile (**9b**) (560 mg, 1.60 mmol), methyl 3-chloro-3-oxopropionate (**10**) (109 mg, 0.80 mmol) and pyridine (126 mg, 1.60 mmol) in abs. CH_2_Cl_2_ (5 mL). The crude product was purified by column chromatography on silica gel (gradient: hexanes/EtOAc, 20:1, then 15:1) to give **11b** as a colourless solid (240 mg, 0.53 mmol, 65%). Mp 63.4 °C; ^1^H NMR (500 MHz, CDCl_3_) δ 1.31–1.35 (m, 10H, 6-H, 7-H, 8-H, 9-H, 10-H), 1.44–1.50 (m, 2H, 11-H), 1.61–1.67 (m, 2H, 5-H), 1.77–1.83 (m, 2H, 12-H), 3.38 (s, 2H, 2-H), 3.75 (s, 3H, OC*H*_3_), 4.00 (t, *J* = 6.5 Hz, 2H, 13-H), 4.14 (t, *J* = 6.8 Hz, 2H, 4-H), 6.98–6.99 (m, 2H, 2'-H), 7.51–7.53 (m, 2H, 3'-H), 7.63–7.64 (m, 2H, 6'-H), 7.68–7.69 (m, 2H, 7'-H) ppm; ^13^C NMR (125 MHz, CDCl_3_) δ 25.7 (C-6), 26.0 (C-11), 28.4 (C-5), 29.15 (C-12), 29.21, 29.33, 29.41, 29.45 (C-7, C-8, C-9, C-10), 41.4 (C-2), 52.4 (O*C*H_3_), 65.7 (C-4), 68.1 (C-13), 110.0 (C-8'), 115.0 (C-2'), 119.1 (*C*N), 127.0 (C-6'), 128.3 (C-3'), 131.3 (C-5'), 132.6 (C-7'), 145.3 (C-4'), 159.8 (C-1'), 166.6 (C-3), 167.0 (C-1) ppm; ATR–FTIR 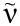: 2927 (m), 2854 (w), 2225 (m), 1735 (s), 1603 (m), 1494 (m), 1249 (s), 1180 (m), 903 (m), 823 (m); ESIMS (*m*/*z*): 474.2 [M + Na]^+^, 452.2 [M + H]^+^; Anal. calcd for C_27_H_33_NO_5_: C, 71.82; H, 7.37; N, 3.10; found: C, 71.66; H, 7.34; N, 3.03; *R*_f_ 0.76 (hexanes/EtOAc 2:1).

### 6-[(4'-cyano-[1,1'-biphenyl]-4-yl)oxy]hexyl 2-cyanoacetate (**13a**)

To a solution of 4'-((6-hydroxyhexyl)oxy)-[1,1'-biphenyl]-4-carbonitrile (**9a**) (100 mg, 338 μmol) in abs. CH_2_Cl_2_ (2.5 mL) were added sequentially a solution of cyanoacetic acid (**12**) (32 mg, 376 μmol) in EtOAc (0.4 mL), a solution of DMAP (12 mg, 98 μmol) in abs. CH_2_Cl_2_ (0.8 mL) and then at 0 °C a solution of dicyclohexylcarbodiimide (77 mg, 376 μmol) in abs. CH_2_Cl_2_ (2.5 mL). The reaction mixture was stirred at rt for 7.5 h, then evaporated under vacuum. The crude product was purified by column chromatography on silica gel (hexanes/EtOAc 12:1) to give **13a** as a colourless solid (72 mg, 190 μmol, 59%). Mp 89.0 °C; ^1^H NMR (500 MHz, CDCl_3_) δ 1.43–1.49 (m, 2H, 6-H), 1.51–1.56 (m, 2H, 7-H), 1.71–1.76 (m, 2H, 5-H), 1.80–1.85 (m, 2H, 8-H), 3.45 (s, 2H, 2-H), 4.01 (t, *J* = 6.4 Hz, 2H, 9-H), 4.23 (t, *J* = 6.3 Hz, 2H, 4-H), 6.98–6.99 (m, 2H, 2'-H), 7.52–7.53 (m, 2H, 3'-H), 7.63–7.64 (m, 2H, 6'-H), 7.68–7.69 (m, 2H, 7'-H) ppm; ^13^C NMR (125 MHz, CDCl_3_) δ 24.7 (C-2), 25.5 (C-6), 25.6 (C-7), 28.3 (C-5), 29.0 (C-8), 66.9 (C-4), 67.8 (C-9), 110.0 (C-8'), 112.9 (C-1), 115.0 (C-2'), 119.1 (*C*N), 127.0 (C-6'), 128.3 (C-3'), 131.3 (C-5'), 132.5 (C-7'), 145.2 (C-4'), 159.6 (C-1'), 162.9 (C-3) ppm; ATR–FTIR 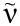: 2941 (m), 2866 (w), 2225 (w), 1746 (m), 1602 (m), 1494 (m), 1249 (m), 1180 (m), 903 (s), 723 (s); ESIMS (*m*/*z*): 385.1 [M + Na]^+^, 363.1 [M + H]^+^; Anal. calcd for C_22_H_22_N_2_O_3_: C, 71.91; H, 6.12; N, 7.57; found: C, 71.44; H, 6.06; N, 7.73; *R*_f_ 0.68 (hexanes/EtOAc 2:1).

### 10-[(4'-cyano-[1,1'-biphenyl]-4-yl)oxy]decyl 2-cyanoacetate (**13b**)

The cyanoacetic ester **13b** was obtained by the same procedure as described above for **13a** from 4'-((10-hydroxydecyl)oxy)-[1,1'-biphenyl]-4-carbonitrile (**9b**) (120 mg, 341 μmol), cyanoacetic acid (**12**) (32 mg, 376 μmol), DMAP (13 mg, 102 μmol), and dicyclohexylcarbodiimide (77 mg, 376 μmol) in abs. CH_2_Cl_2_ (6.5 mL). The crude product was purified by column chromatography on silica gel (hexanes/EtOAc 10:1) to give **13b** as a colourless solid (57 mg, 140 μmol, 41%). Mp 93.2 °C; ^1^H NMR (500 MHz, CDCl_3_) δ 1.32–1.36 (m, 10H, 6-H, 7-H, 8-H, 9-H, 10-H), 1.44–1.50 (m, 2H, 11-H), 1.65–1.71 (m, 2H, 5-H), 1.78–1.83 (m, 2H, 12-H), 3.44 (s, 2H, 2-H), 4.00 (t, *J* = 6.5 Hz, 2H, 13-H), 4.20 (t, *J* = 6.9 Hz, 2H, 4-H), 6.98–6.99 (m, 2H, 2'-H), 7.51–7.53 (m, 2H, 3'-H), 7.63–7.64 (m, 2H, 6'-H), 7.68–7.69 (m, 2H, 7'-H) ppm; ^13^C NMR (125 MHz, CDCl_3_) δ 24.7 (C-2), 25.6 (C-6), 26.0 (C-11), 28.3 (C-5), 29.11 (C-12), 29.21, 29.32, 29.36, 29.40 (C-7, C-8, C-9, C-10), 67.1 (C-4), 68.1 (C-13), 110.0 (C-8'), 113.0 (C-1), 115.0 (C-2'), 119.1 (*C*N), 127.0 (C-6'), 128.3 (C-3'), 131.3 (C-5'), 132.5 (C-7'), 145.2 (C-4'), 159.7 (C-1'), 162.9 (C-3) ppm; ATR–FTIR 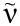: 2928 (m), 2855 (w), 2225 (w), 1747 (m), 1603 (m), 1494 (m), 1250 (m), 1180 (m), 903 (s), 725 (s); ESIMS (*m*/*z*): 457.1 [M + K]^+^, 441.2 [M + Na]^+^, 436.2, 419.2 [M + H]^+^; Anal. calcd for C_26_H_30_N_2_O_3_: C, 74.61; H, 7.22; N, 6.69; found: C, 74.04; H, 7.18; N, 6.54; *R*_f_ 0.58 (hexanes/EtOAc 2:1).

## Supporting Information

File 1 Full experimental procedures and DSC traces of **11b** and **13b**.
