# Supplementary material for: Synthesis and mesomorphic properties of calamitic malonates and cyanoacetates tethered to 4-cyanobiphenyls
Source: Beilstein J Org Chem. 2012 Mar 9;8:371–8. doi: 10.3762/bjoc.8.40 (PMC3326613; doi:10.3762/bjoc.8.40)
Supplement: File 1 — Full experimental procedures and DSC traces of 11b and 13b. [file Beilstein_J_Org_Chem-08-371-s001.pdf]

Supporting Information

for

Synthesis and mesomorphic properties of calamitic malonates and cyanoacetates tethered to 4-cyanobiphenyls

Katharina C. Kress, Martin Kaller, Kirill V. Axenov, Stefan Tussetschläger and Sabine Laschat*

Address: Institut für Organische Chemie, Universität Stuttgart, Pfaffenwaldring 55, 70569 Stuttgart, Germany

Email: Stefan Tussetschläger - stefan.tussetschlaeger@oc.uni-stuttgart.de; Sabine Laschat* - Sabine.Laschat@oc.uni-stuttgart.de

* Corresponding author

Full experimental procedures and DSC traces of 11b and 13b.

Table of Contents

Experimental procedures	S2
DSC studies	S5
References	S6

Experimental procedures

6-bromohexan-1-ol (7a)

A solution of HBr in water (48 wt %, 5.6 mL, 50.8 mmol) was slowly added to a solution of hexan-1,6-diol (**6a**) (5.00 g, 42.3 mmol) in toluene (50 mL). The reaction mixture was heated under reflux for 48 h. After being cooled to rt, the organic and water phases were separated. The organic phase was diluted with Et_2O (30 mL), washed with aqueous NaOH (saturated solution in water, 50 mL), then with aqueous NaCl (saturated solution in water, 50 mL), dried over $MgSO_4$ and evaporated under reduced pressure. The crude product was purified by column chromatography on silica gel (hexanes/EtOAc, 1:1) to give **7a** as a yellow oil (5.45 g, 30.3 mmol, 72%).

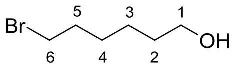

^{1}H NMR (500 MHz, $CDCl_3$) δ 1.22–1.24 (m, 1H, O*H*), 1.36–1.43 (m, 2H, 3-H), 1.44–1.50 (m, 2H, 4-H), 1.56–1.61 (m, 2H, 2-H), 1.85–1.90 (m, 2H, 5-H), 3.14 (t, *J* = 6.7 Hz, 2H, 6-H), 3.64–6.67 (m, 2H, 1-H) ppm; ^{13}C NMR (125 MHz, $CDCl_3$) δ 24.9 (C-3), 27.9 (C-4), 32.5 (C-2), 32.7 (C-5), 33.8 (C-6), 62.8 (C-1) ppm; R_f 0.40 (hexanes/EtOAc, 3:1, potassium permanganate). The obtained spectroscopic data are in accordance with the literature [1].

10-bromodecan-1-ol (7b)

Aqueous HBr (48 wt % solution in water, 3.8 mL, 34.4 mmol) was slowly added to a solution of decan-1,6-diol (**6b**) (5.00 g, 28.7 mmol) in toluene (50 mL). The reaction mixture was heated under reflux for 48 h. After being cooled to rt, the organic and water phases were separated. The organic phase was diluted with Et_2O (30 mL), washed with aqueous NaOH (saturated solution in water, 50 mL), then with aqueous NaCl (saturated solution in water, 50 mL), dried over $MgSO_4$ and evaporated under

reduced pressure. The crude product was purified by column chromatography on silica gel (hexanes/EtOAc, 3:1) to give **7b** as a yellow oil (3.86 g, 16.3 mmol, 57%).

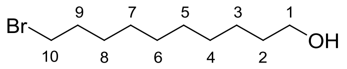

^{1}H NMR (500 MHz, $CDCl_3$) δ 1.27–1.38 (m, 11H, O*H*, 3-H, 4-H, 5-H, 6-H, 7-H), 1.39–1.43 (m, 2H, 8-H), 1.53–1.5 (m, 2H, 2-H), 1.83–1.88 (m, 2H, 9-H), 3.40 (t, *J* = 6.6 Hz, 2H, 10-H), 3.62–6.65 (m, 2H, 1-H) ppm; ^{13}C NMR (125 MHz, $CDCl_3$) δ 25.7 (C-3), 28.1 (C-8), 28.7–29.4 (C-4, C-5, C-6, C-7), 32.7, 32.8 (C-2, C-9), 34.0 (C-10), 63.0 (C-1) ppm; R_f 0.59 (hexanes/EtOAc, 3:1, potassium permanganate). The obtained spectroscopic data are in accordance with the literature [1].

4'-((6-hydroxyhexyl)oxy)-[1,1'-biphenyl]-4-carbonitrile (9a)

A mixture of 6-bromohexan-1-ol (**7a**) (869 mg, 4.80 mmol), 4'-cyano-4-hydroxy-biphenyl (**8**) (936 mg, 4.80 mmol) and potassium carbonate (1.33 g, 9.60 mmol) in abs. acetone (20 mL) was heated under reflux for 24 h. All volatiles were removed under vacuum and the residue was recrystallized from methanol (15 mL, hot filtrated from potassium carbonate) to give **9a** as a colourless solid (959 mg, 3.20 mmol, 68%).

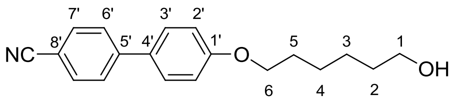

^{1}H NMR (500 MHz, $CDCl_3$) δ 1.42–1.50 (m, 2H, 3-H), 1.50–1.57 (m, 2H, 4-H), 1.59–1.66 (m, 2H, 2-H), 1.80–1.87 (m, 2H, 5-H), 3.67 (t, *J* = 6.6 Hz, 2H, 1-H), 4.01 (t, *J* = 6.6 Hz, 2H, 6-H), 6.99 (d, *J* = 8.6 Hz, 2H, 2'-H), 7.52 (d, *J* = 8.6 Hz, 2H, 3'-H), 7.64 (d, *J* = 8.6 Hz, 2H, 6'-H), 7.90 (d, *J* = 8.6 Hz, 2H, 7'-H) ppm; ^{13}C NMR (125 MHz, $CDCl_3$) δ 25.5 (C-4), 25.9 (C-3), 29.2 (C-5), 32.7 (C-2), 62.9 (C-1), 68.0 (C-6), 110.5 (C-8'), 115.1 (C-2'), 119.1 (*C*N), 127.2 (C-6'), 128.3 (C-3'), 131.3 (C-5'), 132.5 (C-7'), 145.3

(C-4'), 159.7 (C-1') ppm; R_f 0.22 (hexanes/EtOAc, 2:1, potassium permanganate). The obtained spectroscopic data are in accordance with the literature [2].

4'-((10-hydroxydecyl)oxy)-[1,1'-biphenyl]-4-carbonitrile (9b)

A mixture of 10-bromodecan-1-ol (**7a**) (2.37 g, 10 mmol), 4'-cyano-4-hydroxy-biphenyl (**8**) (936 mg, 4.80 mmol) and potassium carbonate (2.76 g, 10 mmol) in abs. acetone (30 mL) was heated under reflux for 24 h. All volatiles were removed under vacuum and the residue was recrystallized from methanol (25 mL, hot filtrated from potassium carbonate) to give **9b** as a colourless solid (2.11 g, 6.00 mmol, 60%).

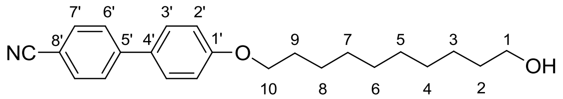

^{1}H NMR (500 MHz, $CDCl_3$) δ 1.21 (t, *J* = 5.2 Hz, 1H, O*H*), 1.32–1.36 (m, 10H, 3-H, 4-H, 5-H, 6-H, 7-H), 1.44–1.50 (m, 2H, 8-H), 1.54–1.58 (m, 2H, 2-H), 1.77–1.83 (m, 2H, 9-H), 3.62–3.65 (m, 2H, 1-H), 3.99–4.01 (m, 2H, 10-H), 6.97–6.99 (m, 2H, 2'-H), 7.52–7.54 (m, 2H, 3'-H), 7.63–7.69 (m, 4H, 6'-H, 7'-H) ppm; ^{13}C NMR (125 MHz, $CDCl_3$) δ 25.7 (C-7), 26.0 (C-8), 29.21 (C-9), 29.35–29.50 (C-3, C-4, C-5, C-6), 32.7 (C-2), 63.0 (C-1), 68.1 (C-10), 110.0 (C-8'), 115.0 (C-2'), 119.1 (*C*N), 127.0 (C-6'), 128.3 (C-3'), 131.3 (C-5'), 132.5 (C-7'), 145.3 (C-4'), 159.7 (C-1') ppm; R_f 0.66 (hexanes/EtOAc, 2:1, potassium permanganate). The obtained spectroscopic data are in accordance with the literature [3].

DSC studies

DSC traces of compounds **11b** and **13b**

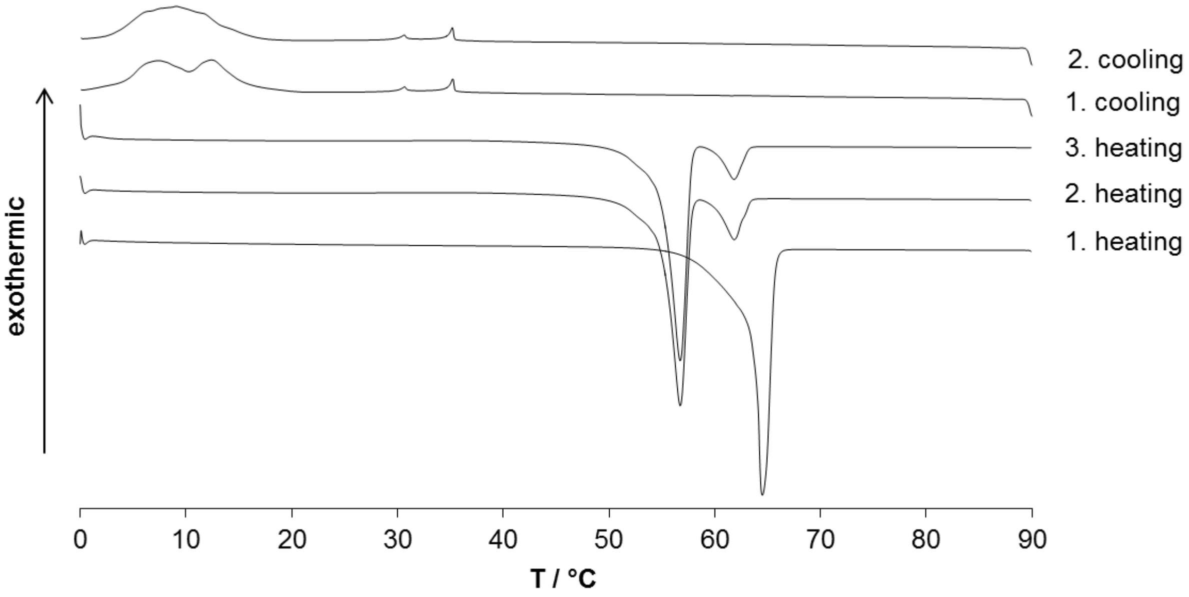

Figure S1: DSC traces of **11b** (heating/cooling rate 5 K/min).

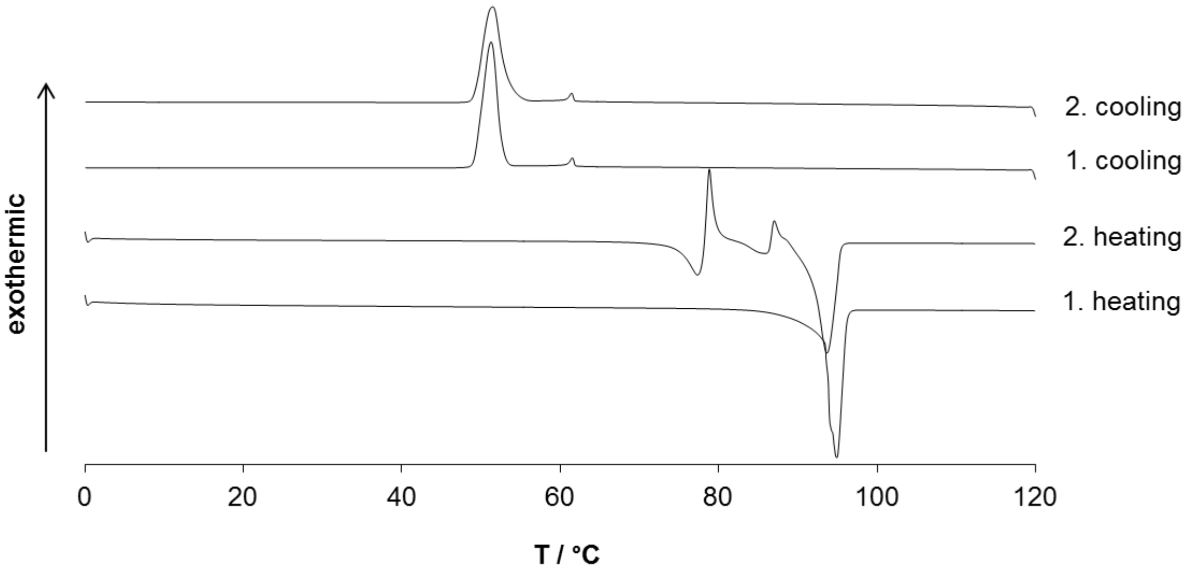

Figure S2: DSC traces of **13b** (heating/cooling rate 5 K/min).

References

1. Chong, J. M.; Heuft, M. A.; Rabbat, F. *J. Org. Chem.* **2000**, *65*, 5837–5838.
2. Sahade, D. A. Polymerizable Liquid Crystal Compound, Polymerizable Liquid Crystal Composition, and Alignment Film. EP 2062882, May 27, 2009.
3. Nakatsuji, S.; Ikemoto, H.; Akutsu, H.; Yamada, J.; Mori, A. *J. Org. Chem.* **2003**, *68*, 1708–1714.
